# Brain-Resident MAIT Cells Infiltrate GL261 Tumors, and Activated MAIT Cell Signatures Are Associated With Improved Outcomes in Glioma

**DOI:** 10.1212/NXI.0000000000200546

**Published:** 2026-01-29

**Authors:** Eleanor M. Eddy, Md Abdullah Al Kamran Khan, Timothy Patton, Davide Moi, Huimeng Wang, Jeremy P. Le, Michael N.T. Souter, Adam G. Nelson, Phoebe M. Dewar, Shihan Li, James McCluskey, Roberta Mazzieri, Riccardo Dolcetti, Zhenjun Chen, Alexander D. Barrow, Alexandra J. Corbett

**Affiliations:** 1 Department of Microbiology and Immunology, University of Melbourne, at the Peter Doherty Institute for Infection and Immunity, Victoria, Australia;; 2 Centre for Innate Immunity and Infectious Diseases, Hudson Institute of Medical Research, Clayton, Victoria, Australia;; 3 Peter MacCallum Cancer Centre, Melbourne, Victoria, Australia; and; 4 Sir Peter MacCallum Department of Oncology, The University of Melbourne, Victoria, Australia.

## Abstract

**Background and Objectives:**

Mucosal-associated invariant T (MAIT) cells are unconventional T cells with emerging roles in antitumor immunity. Their phenotype in the brain and potential role in immunity to gliomas—including lower-grade (WHO grades I and II) and higher-grade gliomas such as grades III and IV (glioblastoma)—remain poorly defined.

**Methods:**

We assessed the role of MAIT cells in glioma using publicly available transcriptomic data from patient cohorts. We then characterized MAIT cells in the mouse brain using flow cytometry and assessed their impact on survival and on other immune cells in the murine GL261 model of high-grade glioma. We tested previously developed methods to activate and expand MAIT cells in mice for their effect on brain MAIT cells.

**Results:**

Analysis of The Cancer Genome Atlas revealed an association between a gene signature of activated, but not naïve, MAIT cells and improved survival in patients with grade III glioma. In mice, MAIT cells were predominantly brain-resident and infiltrated GL261 tumors where they produced IL-17 and IFN-γ. Notably, MAIT cell–deficient *Mr1*^−/−^ mice displayed reduced survival after GL261 tumor induction, suggesting a protective role for MAIT cells in higher grade gliomas. Injection of MAIT antigen and adjuvants expanded brain-resident MAIT cells, but expansion of MAIT cells alone prior to GL261 tumor induction did not significantly alter survival.

**Discussion:**

Overall, this study supports a protective role for a population of brain-resident MAIT cells in glioma and highlights their potential involvement in immune surveillance of the CNS. Our findings also lay a foundation to explore the therapeutic modulation of MAIT cells in the brain.

## Introduction

Gliomas are primary brain tumors classified into grades I–IV based on histopathologic and molecular features, with glioblastoma (GBM) representing the most aggressive form. GBM is incurable, with a median survival time of 15 months after diagnosis.^1^ Standard treatment, combining surgical resection, radiotherapy, and adjuvant chemotherapy,^2^ is ineffective in preventing recurrence. To date, immunotherapies have demonstrated poor efficacy in GBM due to an immunosuppressive tumor microenvironment (TME), restricted leukocyte infiltration of tumors cause by the blood-brain barrier, and limited T-cell antigen targets.^3^ The detailed characterization of T cells within the brain and their response to tumors is warranted to expand the scope of therapeutic options for GBM.

Mucosal-associated invariant T (MAIT) cells are unconventional T cells with an emerging role in antitumor immunity.^4^ The MAIT T-cell receptor (TCR) recognizes microbial metabolite antigens, the most potent being 5-(2-oxopropylideneamino)-6-D-ribitylaminouracil (5-OP-RU), presented by the major histocompatibility complex (MHC) class I–related molecule-1 (MR1).^5,6^ MR1–5-OP-RU tetramers^5,7^ have enabled the specific identification of MAIT cells, which are abundant in the circulation and mucosal tissues in humans and show a similar distribution, but lower abundance, in mice.^8,9^ Although MAIT cells have been identified in the brain,^10,11^ they remain incompletely characterized at this site. Adjuvant-induced MAIT cell expansion in the brain has not been previously assessed.

MAIT cells have innate-like effector functions driven by expression of the transcription factor promyelocytic leukemia zinc finger (PLZF).^12^ In both mice and humans, circulating MAIT cells predominantly express IFN-γ, whereas mucosal tissue–resident MAIT cells express IL-17 and genes associated with tissue repair.^13,14^ Thus, the functional states of MAIT cells are shaped by tissue-specific cues. MAIT cell responses to noninfectious signals, such as those encountered during wound healing and malignancy, and their role in nonmucosal tissues, such as the brain, are emerging.

TRAV1-2 transcripts, suggesting MAIT cell presence, were identified within human brain tumors in 2008.^15^ More recently, a population of meningeal MAIT cells was found to produce antioxidants that restrict neuroinflammation and maintain meningeal barrier integrity.^10^ Supporting a specialized role for MAIT cells in neurosurveillance, *MR1* polymorphisms affecting *MR1* gene expression have been associated with the development of cerebral tuberculosis,^16^ and proinflammatory MAIT cells have been shown to infiltrate white matter lesions in patients with multiple sclerosis, particularly in regions with upregulated expression of MR1.^17,18^ MAIT cells appear to provide protection in the experimental autoimmune encephalomyelitis mouse model of multiple sclerosis,^19^ whereas mouse models of Alzheimer disease so far yield apparently contradictory findings.^20,21^ Evidence of clinical relevance to brain tumors has been documented more recently; higher transcriptional expression of *MR1* has been observed in high-grade gliomas and is associated with reduced survival.^22^

In this study, we explored MAIT cell transcriptional signatures in the Cancer Genome Atlas (TCGA) data sets and characterized MAIT and other T cells in the mouse brain and in the GL261 model of GBM. Our findings provide insights into brain-resident MAIT cells and suggest a potential role in the immune response to glioma. We showed that systemic MAIT cell boosting is effective in expanding MAIT cell populations within the brain, enabling their detailed phenotypic characterization and providing tools for their further assessment as a therapeutic target in glioma and neurologic diseases.

## Methods

### Mice

C57BL/6 (wild-type, WT) and *Mr1*^−/−^ mice^23^ were bred and housed under specific pathogen-free conditions at the Biological Research Facility of the Doherty Institute (Melbourne, Australia) or the Peter MacCallum Cancer Centre (Melbourne Australia), with a 12-hour day/night cycle, 40%–70% humidity, and a temperature of 19–22 °C. Mice were 6–12 weeks old at the beginning of experiments and at least 7 weeks old prior to intracranial injections and were acclimatized for at least 7 days after transfer between facilities prior to any procedure. Intracranial injection experiments were performed using female mice for technical and ethical reasons. Researchers randomly assigned mice to treatment groups. Each mouse represented an experimental unit, with none excluded. Sample size was determined by logistical considerations of each experiment. Methods are reported in accordance with the Animal Research: Reporting of In Vivo Experiments (ARRIVE) guidelines.^24^

### Statistical Analysis

In silico analyses were performed using R version 4.4.2, and group means were compared by the nonparametric Wilcoxon signed-rank test. Prism GraphPad software (version 10, La Jolla, CA) was used for statistical comparisons, including 2-tailed unpaired *t* tests, Analysis of Variance (ANOVA), or Mantel-Cox tests, as indicated in figure legends. Data show mean ± standard error of the mean (SEM), unless otherwise stated.

### Standard Protocol Approvals and Registrations

All procedures on mice were performed in accordance with Australian Government guidelines after approval by the Animal Ethics Committees of the University of Melbourne (#10201) or Peter MacCallum Cancer Centre (#2022-15 and #2023-30).

### Data Availability

The original data presented in the study are included in the article/supplemental material. Inquiries should be directed to the corresponding authors.

## Results

### A Transcriptional Signature of Activated MAIT Cells Is Associated With Improved Survival in Patients With Glioma

**Figure 1:**
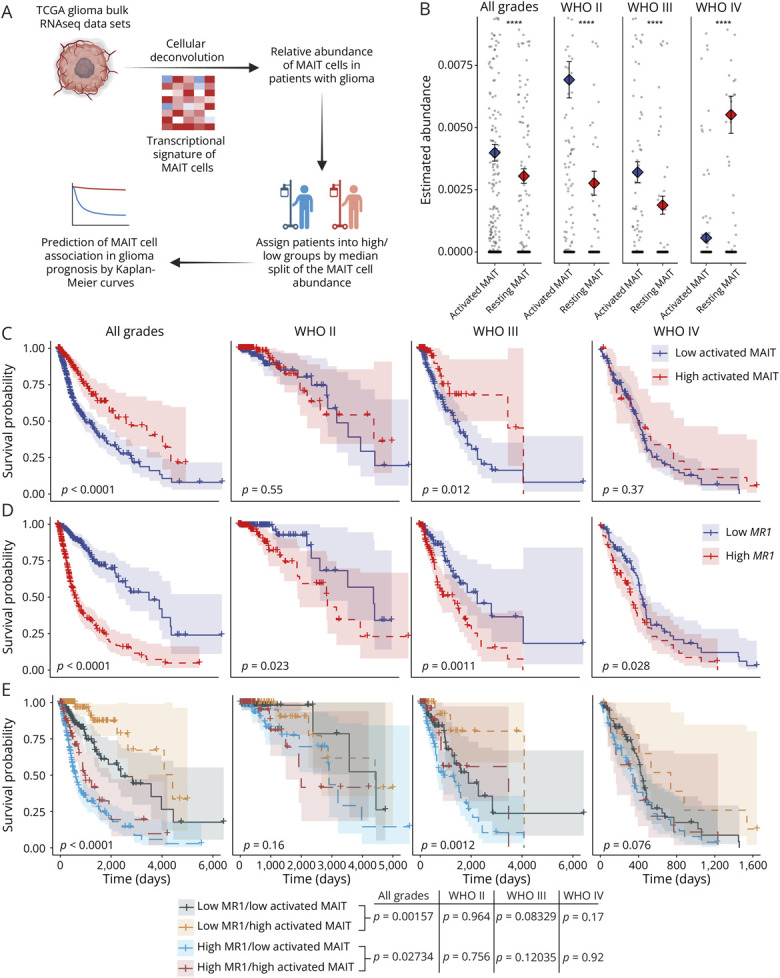
Activated MAIT Cell Signatures Are Associated With Improved Survival in Patients With Glioma (A) Computational workflow to assess the association of MAIT cells in TCGA glioma patient data sets. Created in BioRender. Corbett, A. (2026) BioRender.com/8m6317t. (B) Comparison of the estimated abundance of the activated and resting MAIT cell gene signatures across different glioma grades (mean ± SEM values are presented by dot and whiskers; each dot represents the datapoint from an individual patient; ****p value <0.0001, Wilcoxon signed-rank test was used to compare the group means). (C-D) Kaplan Meier (KM) curves representing the prognostic implications of (C) activated MAIT cells and (D) *MR1* abundance, in different glioma grades. (E) KM curves showing the combined association of *MR1* abundance and activated MAIT cells in glioma prognosis (*p* values within the KM curves represent the global *p* value of the comparisons between the groups; *p* values of the pairwise comparisons are provided below the KM curves). MAIT = mucosal-associated invariant T; SEM = standard error of the mean; TCGA = The Cancer Genome Atlas.

MAIT cells can contribute to antitumor immunity,^25,26^ and intratumoral *MR1* expression has been linked to poor survival in patients with glioma.^22^ However, the potential roles of MAIT cells in glioma remain poorly understood. To explore possible associations between MAIT cell function and glioma outcomes, we generated transcriptional signatures for both resting and activated MAIT cells,^27^ as described in eMethods, using our previously published data set^28^ (Figure 1A, eFigure 1A). These signatures were enriched for canonical markers such as *TRAV1-2* and *RORC*, along with distinctive genes including *TNFRSF9*, *FASLG*, *SLC4A10*, and *CCR2* (eMethods, eFigure 1, C and D). Cellular deconvolution revealed both “resting” and “activated” MAIT cell signatures in glioma patient transcriptomes (Figure 1B). There was a trend toward a higher abundance of activated compared with resting MAIT cell signatures in grade II and III gliomas, whereas grade IV gliomas had relatively higher resting MAIT cells (Figure 1B). Patients with glioma exhibiting higher levels of the activated MAIT cell signature showed enrichment of functional pathways associated with T-cell proliferation and activation, as well as TCR, IL-17, tumor necrosis factor (TNF), and Toll-like receptor signaling (eFigure 1B). Of interest, higher levels of the activated MAIT cell signature were associated with an increased probability of survival, especially in patients with grade III glioma (Figure 1C). By contrast, higher *MR1* expression was associated with a reduced probability of survival, consistent with previous findings (Figure 1D).^22^ Regardless of *MR1* expression, a higher activated MAIT cell signature was associated with better patient survival outcomes (Figure 1E). Multivariate analyses showed that activated MAIT cells and *MR1* expression were each associated with patient outcomes independently of other T cell or natural killer (NK) cell subsets and HLA-I molecules, respectively (eFigure 1, E and F). Overall, these findings suggest a positive link between MAIT cell activity and survival in glioma.

### MR1-Deficient Mice Have Reduced Survival Time After the Induction of GL261 Tumors

**Figure 2:**
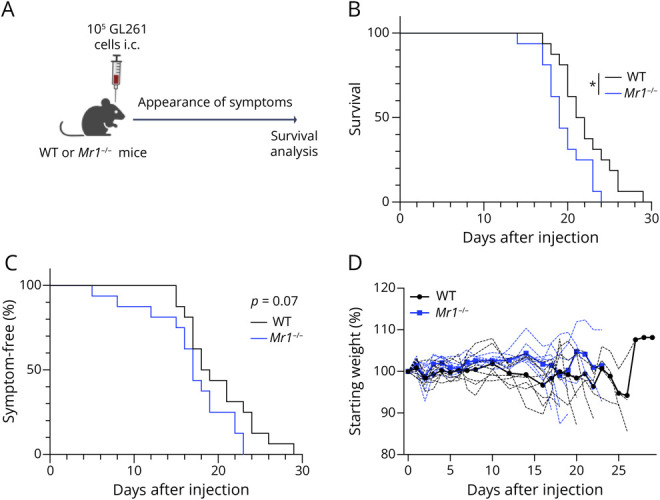
Survival After Intracranial Tumor Development Is Reduced in *Mr1−/−* Mice Compared With WT Mice (A) Schematic: WT and *Mr1*^−/−^ mice were injected intracranially with 10^5^ GL261 cells and monitored for survival analysis. Created in BioRender. Corbett, A. (2026) BioRender.com/8m6317t (B) Kaplan-Meier analysis of survival, showing a median survival time of 21.5 days (WT) and 19 days (*Mr1*^−/−^). *p < 0.05 (Mantel-Cox test). (C) Symptom-free survival. (D) Mouse weights after injection of GL261 cells. Bold line represents the average; dotted lines represent individual animals. N = 16 mice per group, pooled from 2 experiments.

Our computational analysis prompted us to examine the presence and role of MAIT cells in glioma experimentally. We used an orthotopic mouse model of GBM with GL261 cells, a C57BL/6-derived glioma cell line.^29^ C57BL/6 (WT) and *Mr1*^−/−^ mice, which lack MAIT cells,^30^ were injected intracranially with GL261 cells and culled based on humane end points (Figure 2A, eMethods). In *Mr1*^−/−^ mice, survival time was reduced by approximately 10% compared with WT mice (Figure 2B). Furthermore, while not statistically significant, symptoms appeared earlier in *Mr1*^−/−^ mice than in WT mice (median time to symptoms: 17 vs 18.5 days, *p* = 0.07) (Figure 2C). Weight loss was similar between mouse strains (Figure 2D), and tumor size did not correlate with survival time (eFigure 2A). There was no significant difference in tumor mass between mouse strains at the end point (eFigure 2B). In separate experiments in which tumors were collected at day 18, tumor mass (eFigure 2C) and relative burden (percentage of brain mass) (eFigure 2D) were comparable between WT and *Mr1*^−/−^ mice, suggesting that tumor size does not dictate survival time.

### MAIT and Other Unconventional T Cells Are Tissue-Resident Cells in the Healthy Mouse Brain

**Figure 3:**
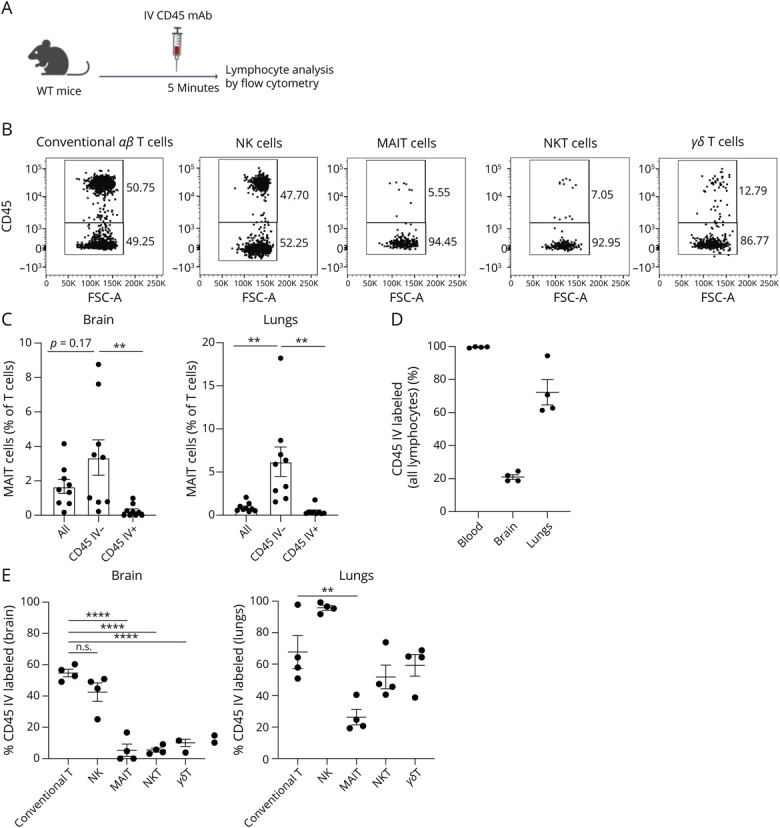
MAIT Cells Are Predominantly Tissue Resident in the Mouse Brain (A) Schematic: 5 minutes prior to sacrifice and organ harvest, naive 8-week-old C57BL/6 mice were injected IV with 1 μg of PE-conjugated or FITC-conjugated anti-CD45.2. Created in BioRender. Corbett, A. (2026) BioRender.com/8m6317t. (B) Representative plots showing CD45 IV labeling of conventional T cells, NK cells, MAIT cells, NKT cells, and γδ T cells in the brains of healthy WT C57BL/6 mice. (C) MAIT cell frequency in the brain and lung tissue of all αβ T cells and of tissue-resident (CD45 IV-labeled) αβ T cells in the brain and lungs. (D) Percentage of CD45 IV-labeled lymphocytes in the brain, lung, and blood of WT C57BL/6 mice. (E) Percentage of CD45 IV-labeled indicated cell populations in the brain and lungs of WT C57BL/6 mice. Data represent the mean ± SEM of 6–9 mice, representative of 6 independent experiments. In (C and E), ****p < 0.0001 and **p < 0.01; 1-way ANOVA. All others insignificant (*p* > 0.05). MAIT = mucosal-associated invariant T; NKT =natural killer T.

We next sought to characterize the frequency and phenotype of MAIT cells within the healthy mouse brain. We examined the brains of naïve WT mice via flow cytometry, distinguishing between circulating and resident MAIT cells by CD45 mAb injected IV before euthanasia (Figure 3, A and B). MAIT cells represented approximately 1.7% of αβ T cells in the brain (Figure 3B) and approximately 0.9% of αβ T cells in the lungs (Figure 3C, eFigure 3A), consistent with previous studies.^8,10,23^ CD45 IV labeling revealed a greater proportion of tissue-resident lymphocytes in the brain than in the lungs (Figure 3D), with half of conventional T cells and NK cells in the brain IV labeled (Figure 3E). Unconventional T cells, including MAIT, natural killer T (NKT), and γδ T cells, were largely unlabeled, indicating tissue residency (Figure 3E, eFigure 3B), a trend recapitulated by MAIT cells in the lungs (Figure 3E). Most of the brain MAIT cells also expressed the tissue residency marker CD103 (eFigure 4A). Expression of CD69 was similar between T-cell subsets, except for NKT cells, which were approximately 70% CD69^+^ (eFigure 4B). PD-1 expression was variable in MAIT and other T cells (eFigure 4C). These results show that MAIT and other unconventional T cells are predominantly tissue resident in the mouse brain and may play a unique role in immune surveillance of the CNS.

### MAIT Cells Infiltrate and Respond to GL261 Brain Tumors

**Figure 4:**
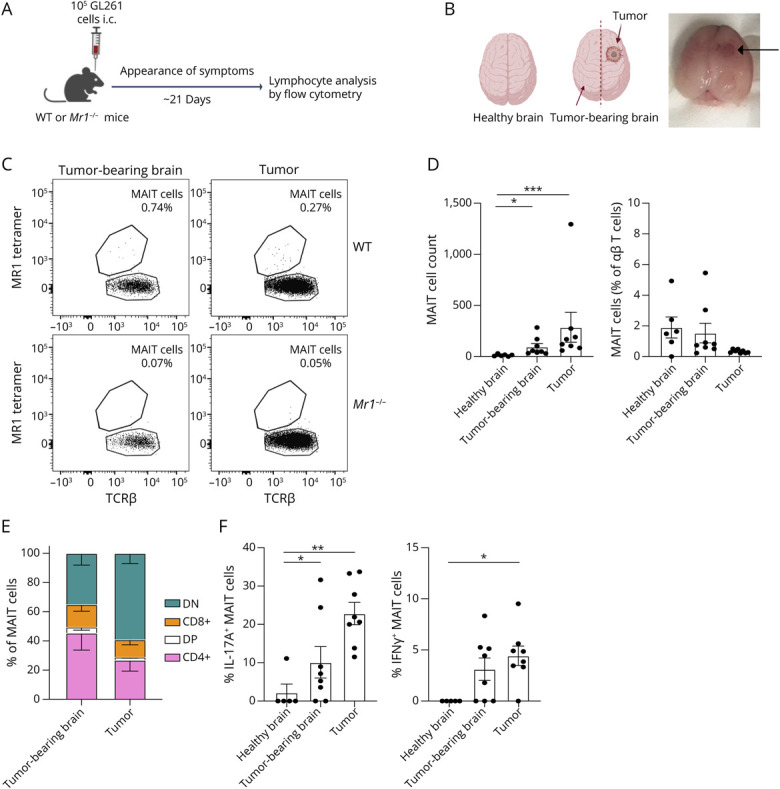
MAIT Cells Infiltrate GL261 Tumors (A and B) Diagram summary of the experimental method and tissue processing for analysis. C57BL/6 (WT) mice were intracranially injected with 10^5^ GL261 tumor cells, and survival and development of symptoms were assessed until mice exhibited end point symptoms and were euthanized. Photograph in B shows the brain from 1 tumor-bearing mouse. Arrow indicates the tumor. Created in BioRender. Corbett, A. (2026) BioRender.com/8m6317t. (C) Representative flow cytometry plots of αβ T cells, with gated MAIT cells, in tumor-bearing brains (contralateral hemisphere to brain tumor) and tumor tissues of WT and *Mr1*^−/−^ mice. (D) Absolute numbers and relative abundance (as a percentage of total αβ T cells) of MAIT cells in WT mice. Data points represent individual mice, representative of 4 independent experiments. (E) Co-receptor distribution and (F) IL-17 and IFN-γ expression by MAIT cells in healthy brains, tumor-bearing brains, and tumor tissues 18 days after injection. Bars represent mean ± SEM; data points represent individual mice. *p < 0.05, **p < 0.01, ***p < 0.001 (1-way ANOVA with Tukey multiple comparisons). MAIT = mucosal-associated invariant T.

We found that GL261 cells can present antigen via MR1 to a MAIT reporter cell line (eFigure 5). Next, we assessed the abundance and phenotype of GL261 tumor–infiltrating MAIT cells compared with those in the contralateral hemisphere (“tumor-bearing brain”) and in brains of naïve mice (Figure 4, A and B). Strikingly, the number of MAIT cells in excised tumors was substantially higher than that in the entire brains of healthy naïve mice (Figure 4, C and D). Furthermore, tumor-bearing brains (contralateral hemisphere) showed greater numbers of MAIT cells compared with the whole healthy brains (Figure 4D). Thus, MAIT cells infiltrate into GL261 tumors and increase in number in the contralateral brain tissue. However, the MAIT cell frequency (percentage of αβ T cells) did not increase compared with healthy naïve brains (Figure 4, C and D). We next characterized the phenotype of tumor-infiltrating MAIT cells. Tumors contained a high proportion of CD4^−^CD8^−^ (DN) MAIT cells, as is seen in other tissues,^8,31^ and CD4^+^ MAIT cells, while the tumor-bearing brain had relatively more CD4^+^ MAIT cells (Figure 4E). Approximately half of MAIT cells in both the tumor-bearing brain and tumor tissue expressed CD69 (eFigure 6A), with a small proportion expressing PD-1, which appeared lower than in healthy brains (compare eFigure 6B and eFigure 4C).

In naïve mice, MAIT cells can be characterized as MAIT1 (IFN-γ producing) or MAIT17 (IL-17A producing) based on the expression of the transcription factors Tbet and RORγt, respectively.^8^ The proportion of each subset differs between tissues^8,32^ but has not been reported previously in the brain. Thus, we assessed the expression of Tbet and RORγt in brain MAIT cells via intracellular staining. We found that healthy mice had a mixed population of MAIT cells (eFigure 6C) while tumors and tumor-bearing brains showed an increase in the proportion of MAIT cells expressing RORγt^+^ (eFigure 6C). We analyzed IFN-γ and IL-17A production by intracellular staining without additional stimulation and found that subsets of MAIT cells in tumors and contralateral tissue produced each cytokine (Figure 4F). Despite representing only approximately 0.5% of tumor-infiltrating T cells (Figure 4D), MAIT cells accounted for almost 10% of IL-17–producing αβ T cells and almost 1% of IFN-γ–producing αβ T cells in GL261 tumors (eFigure 6, D and E). Overall, these data demonstrate that MAIT cells infiltrate GL261 intracranial tumors and drive local production of IL-17 and, to a lesser extent, IFN-γ.

### Tumor-Infiltrating T Cells Are Minimally Altered in *Mr1*^−/−^ Mice Compared With WT Mice

MAIT cells can influence the tissue infiltration and activation of other T cells in tumor settings.^25^ Thus, we next examined whether the presence or absence of MAIT cells affected the infiltration and phenotype of other αβ T cells in WT and *Mr1*^−/−^ mice bearing GL261 tumors. We found that numbers of non-MAIT αβ T cells in the tumor-bearing brain were similar between WT and *Mr1*^−/−^ mice (eFigure 7A). The non-MAIT αβ T–cell population was predominantly CD4^+^ and, unlike MAIT cells (Figure 4E), this was largely consistent between the tumor-bearing brain and tumor tissue (eFigure 7B). We found a significant increase in Tbet expression and greater proportions of non-MAIT αβ cells producing IFN-γ in tumor-bearing brains and tumor tissues compared with naïve brains, but no significant differences between WT and *Mr1*^−/−^ strains (eFigure 7, C and D). Unlike MAIT cells, very few non-MAIT αβ T cells expressed RORγt (eFigure 7C).

To understand the impact of MAIT cells on T-cell subsets, we separately analyzed conventional T cells and NKT cells. Conventional T-cell numbers in the tumor-bearing brain and tumor tissue were comparable in WT and *Mr1*^−/−^ mice (eFigure 7E). While NKT cells trended toward increased numbers in tumors compared with tumor-bearing brains (eFigure 7F), there was no difference in their frequency between WT and *Mr1*^−/−^ mice (eFigure 7G). These findings suggest that, like MAIT cells, NKT cell recruitment is proportional to αβ T cells, and the absence of MAIT cells does not affect NKT recruitment. We found that in WT mice, but not *Mr1*^*−/−*^ mice, the proportion of conventional T cells expressing CD69 was higher in tumor tissues than in tumor-bearing brains, suggesting enhanced activation or tissue residency (eFigure 7H). Conventional T-cell expression of PD-1 trended toward an increase in tumor tissue in both WT and *Mr1*^−/−^ mice (eFigure 7I). CD69 was expressed by most NKT cells (eFigure 7J) while PD-1 was only expressed by a small proportion (∼2%) of NKT cells (eFigure 7K), with no significant difference in the expression of either marker between tumor-bearing brains and tumor tissues or between WT and *Mr1*^−/−^ mice (eFigure 7, J and K). Together, these data suggest that MAIT cells do not regulate the recruitment of other αβ T cells to the tumor site but may affect T-cell activation or tissue residency.

### MAIT Cells Can Be Boosted in the Brain Using Systemic Vaccination Protocols

**Figure 5:**
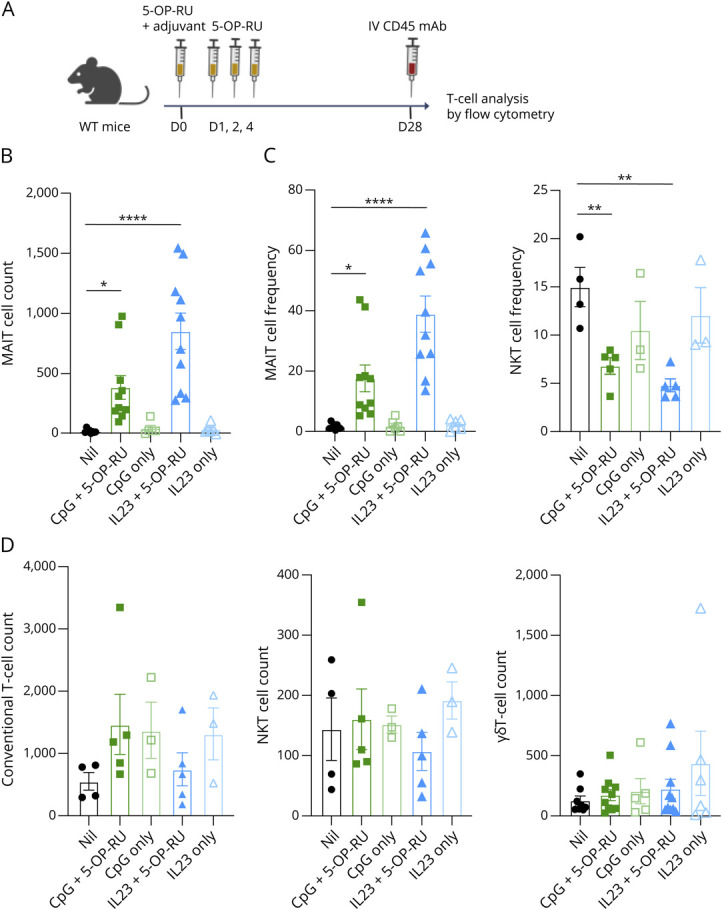
MAIT Cells Can Be Expanded in the Mouse Brain (A) Diagrammatic representation of the experimental method. 8-week-old female C57BL/6 mice were treated with either CpG or IL-23 plasmid with or without subsequent injections of 5-OP-RU, and lymphocytes in the brain were analyzed after 28 days, with PE-labeled CD45 antibody injected 5 minutes before sacrifice to label and exclude circulating cells. Created in BioRender. Corbett, A. (2026) BioRender.com/8m6317t. (B) Absolute counts of MAIT cells; (C) relative frequencies of tissue-resident (CD45.2 IV negative) MAIT (MR1-tetramer positive) and NKT (CD1d-tetramer positive) cells as a proportion of total αβ T cells in the mouse brain; and (D) absolute counts of non-MAIT αβ T, NKT, and γδ T cells. Data points represent individual mice pooled from N = 2 independent experiments. Bars represent mean ± SEM. *p < 0.05, **p < 0.01, ****p < 0.0001 (one-way ANOVA with multiple comparisons). MAIT = mucosal-associated invariant T.

The expansion of MAIT cells in vivo facilitates their analysis in mice, where steady-state numbers are lower than in humans, and MAIT “vaccination” can enhance antibacterial and antitumor immunity in the lungs, liver, and other tissue sites.^25,26,32-34^ With T cells in the brain only recently described, how T cells traverse the blood-brain barrier remains incompletely understood.^35^ To determine whether MAIT cells could be expanded in the brain, we used our previously established systemic vaccination strategies.^32,33^ WT C57BL/6 mice were treated with CpG or IL-23, alone or together with MAIT antigen 5-OP-RU, and then rested for 28 days, before analysis of CD45 IV-negative cells (Figure 5A). Consistent with observations in other tissue sites,^23,32,33,36^ MAIT cell numbers and frequencies were significantly increased after injection of 5-OP-RU with either CpG or IL-23, but not by CpG or IL-23 alone (Figure 5, B and C). As expected, conventional T cells, NKT cells, and γδ T-cell numbers were not altered by these vaccination strategies (Figure 5D), although the NKT cell frequency was reduced (Figure 5C), likely due to the increase in MAIT cells. The expanded noncirculating MAIT cell population after boosting with CpG plus 5-OP-RU expressed Tbet, while IL-23 plus 5-OP-RU resulted in a predominantly RORγt^+^ MAIT cell population (eFigure 8, A and B), demonstrating that MAIT cells can be readily manipulated in the brain with targeted expansion protocols.

Despite significant expansion of MAIT cells in the brain, neither protocol affected overall survival, symptom-free survival, or weight loss after subsequent challenge with GL261 tumor cells (eFigure 9, A–G). While not protective in these highly aggressive models, these data demonstrate that substantial and specific expansion of MAIT1 or MAIT17 populations can be achieved in the brain without obvious detrimental effect, thus highlighting an intriguing new avenue for further therapeutic investigation in the context of MAIT cell surveillance of the brain.

### MAIT Cell Expansion Results in Phenotypic and Functional Changes in MAIT and Non-MAIT T Cells in the Brain

**Figure 6:**
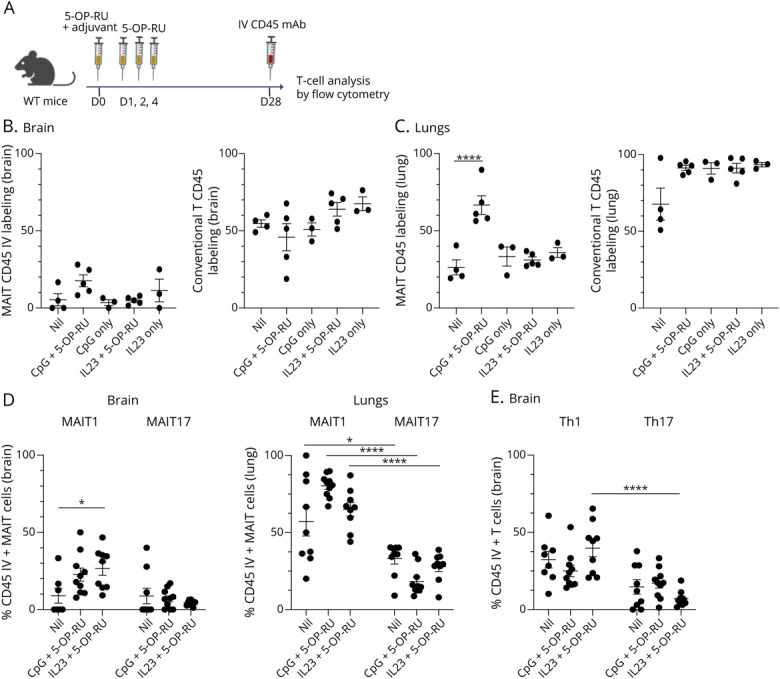
Expanded MAIT Cells in the Brain Reside Within Brain Tissue (A) WT C57BL/6 mice were injected with CpG or IL-23 plasmid, with or without subsequent injections of 5-OP-RU to boost MAIT cells, as shown in Figure 5. Mice were injected IV 5 minutes prior to sacrifice with PE-labeled or FITC-labeled anti-CD45.2, and αβ T cells in brains and lungs were analyzed by flow cytometry. Created in BioRender. Corbett, A. (2026) BioRender.com/8m6317t. (B) Percentage of CD45 IV-labeled MAIT and conventional T cells in the brains and (C) in the lungs of C57BL/6 mice untreated (Nil) or vaccinated with CpG or IL-23 ± 5-OP-RU. (D) Percentage of CD45 IV-labeled MAIT1 and MAIT17 cells, defined by expression of Tbet and RORγt, in the brains and lungs. (E) Percentage of CD45 IV-labeled Th1 (Tbet^+^) and Th17 (RORγt^+^) conventional T cells in the brains. Bars represent the mean ± SEM of 4–5 mice, representative of 2 independent experiments. *p < 0.05, ****p < 0.0001 (2-way ANOVA with multiple comparisons). MAIT = mucosal-associated invariant T.

We next examined MAIT cells and other lymphocytes 28 days after MAIT expansion (Figure 6A). As in naïve mice, most MAIT cells in the brain after expansion protocols were tissue resident (CD45 IV unlabeled; Figure 6B, eFigure 10A). In the lungs, 5-OP-RU plus CpG boosting resulted in an increase in IV-labeled circulatory MAIT cells, while there was no significant increase in the brain (Figure 6, B and C). A higher percentage of conventional T cells than MAIT cells were IV labeled in brains and lungs of boosted mice, like in naïve mice (Figure 6, B and C). Consistent with reported differences in tissue residency,^37^ more MAIT1 cells (Tbet^+^) than MAIT17 cells (RORγt^+^) in the brain and lungs were IV labeled (Figure 6D). Similar characteristics were evident for conventional T cells with more Th1 than Th17 cells IV labeled (Figure 6E). Notably, in the brain, most MAIT cells were unlabeled regardless of treatment or MAIT1/MAIT17 phenotype, although boosting increased IV labeling in MAIT1 cells, which reached significance in mice treated with IL-23 + 5-OP-RU (Figure 6D). Thus, while MAIT cells in the brain are mostly tissue resident, MAIT1 cells are more likely to be circulating than MAIT17 cells and the expansion of MAIT cells in the brain results in an increase in the proportion of MAIT1 cells accessible to the circulation.

As boosted MAIT cells in the brain were predominantly tissue resident, we further characterized the CD45 IV-negative MAIT cells in the brain. As expected, they expressed the master transcription factor PLZF (eFigure 10B), which was higher in MAIT17 than in MAIT1 cells in both naïve and CpG plus 5-OP-RU–treated mice, but this difference was less apparent in IL-23 plus 5-OP-RU–treated mice (eFigure 10B). CD44 expression was also higher on naïve MAIT17 cells but decreased after treatment with 5-OP-RU plus IL-23 (eFigure 10C). The proportion of CD4^−^CD8^−^double-negative (DN) MAIT cells increased after IL-23 + 5-OP-RU vaccination, with concomitant reduction in CD8^+^ MAIT cells (eFigure 10D). Non-MAIT αβ T cells showed a similar change, which was not observed with IL-23 alone. Owing to the low MAIT cell numbers, NK1.1 expression was not assessed in naïve mice, while approximately 50% of MAIT cells expressed NK1.1 in boosted mice. By contrast, NK1.1 was expressed by approximately 20% of conventional T cells and NKT cells and this was not significantly altered by MAIT cell boosting protocols (eFigure 10E). In untreated mice, some MAIT cells expressed granzyme B, TNF, and IFN-γ and approximately 20% produced IL-17 (eFigure 11A). Expansion toward either MAIT1-dominant (CpG) or MAIT17-dominant (IL-23) populations did not enhance cytokine or granzyme production, when measured directly ex vivo without restimulation; instead TNF and IL-17 production decreased (eFigure 11A). GzmB-producing and IL-17-producing MAIT cells also decreased in the lungs while cytokine production by non-MAIT αβ T cells was unchanged (eFigure 11, B and C). Notably, MAIT cells represented around half of the IL-17–producing αβ T cells without prior boosting and approximately 20% of cells producing TNF or GzmB (eFigure 12). This increased further after IL-23 + 5-OP-RU boosting (eFigure 12). Thus, while expanded MAIT cells in the brain are not significantly activated after 28 days, with only small proportions of MAIT cells producing inflammatory cytokines, they account for a relatively large proportion of cytokine-producing αβ T cells.

### MAIT Cell Expansion Alters GL261 Tumor–Infiltrating T Cells

**Figure 7:**
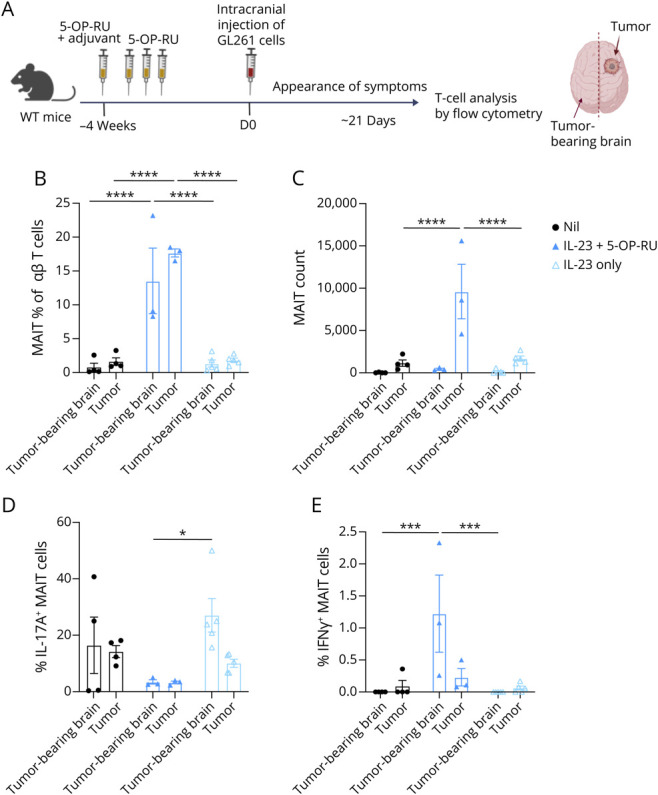
MAIT Expansion IL-23 and 5-OP-RU Alters the MAIT Cell Response to GL261 Tumors (A) C57BL/6 mice were vaccinated with IL-23 plasmid via hydrodynamic injection, with or without 5-OP-RU, and rested for 28 days before intracranial injection with 10^5^ GL261 cells. Created in BioRender. Corbett, A. (2026) BioRender.com/8m6317t. (B) MAIT frequency and (C) absolute counts in the tumor-bearing brains and tumor tissues, at the time of mice exhibiting end point symptoms (approx.18–21 days after intracranial injection). (D) IL-17A and (e) IFN-γ production by MAIT cells in the tumor-bearing brain and tumor tissue. Bars represent mean ± SEM, N = 3–5 per group. *p < 0.05, ***p < 0.001, ****p < 0.0001 (2-way ANOVA). MAIT = mucosal-associated invariant T; SEM = standard error of the mean.

To determine whether boosting MAIT cells affected the broader T-cell response to GL261 tumors, we compared mice boosted 28 days prior to intracranial injection of GL261 cells with unboosted mice (Figure 7A). Analysis of TILs at the humane end point showed an increase in the absolute number and frequency of MAIT cells in both tumor-bearing brains and tumor tissues in mice preboosted with IL-23 plus 5-OP-RU, with MAIT cells comprising up to 20% of total αβ T cells (Figure 7, B and C). By contrast, intratumoral MAIT cell frequencies were not elevated in the CpG + 5-OP-RU–boosted mice (data not shown). Thus, for further analysis, we compared mice treated with 5-OP-RU plus IL-23 with unboosted control mice. The proportion of IL-17–producing MAIT cells in the tumor-bearing brain was lower in mice treated with IL-23 + 5-OP-RU compared with those treated with IL-23 alone or nonboosted mice (Figure 7D), with a small but significant increase in IFN-γ production (Figure 7E). Similarly, the proportion of MAIT cells producing GzmB or TNF (eFigure 13, A and B) was generally low across treatment groups and further decreased in the tumor tissue compared with the tumor-bearing brain. Tumor infiltration by non-MAIT αβ T cells was highest in the IL-23–treated mice and lower in mice treated with IL-23 plus 5-OP-RU (eFigure 13C). No significant differences were observed in IL-17A, IFN-γ, or GzmB production of non-MAIT αβ T cells (eFigure 13D). TNF expression appeared to decrease in all groups in the tumor tissue, but not significantly (eFigure 13C). Together, these data show phenotypic changes in both MAIT and non-MAIT αβ T cells in GL261 tumors compared with the tumor-bearing brains, and indicated that, although MAIT cell numbers are increased in the tumor tissue, their cytokine response appears to be dampened in the tumors.

## Discussion

In this study, we examined MAIT cells in patients with glioma using cellular deconvolution^38^ and characterized their phenotype and function in a mouse model of GBM. Consistent with a previous report,^22^ *MR1* expression was associated with poor survival in the glioma TCGA data sets,^39^ suggesting a detrimental role in tumor progression. However, even in the presence of high levels of *MR1*, a gene signature of activated MAIT cells was positively associated with improved survival, particularly at WHO grades II and III, suggesting that MAIT cells may play a role in antitumor immunity, likely exerting their action at earlier stages of disease. The shorter survival of MAIT cell–deficient *Mr1*^−/−^ mice after induction of GL261 tumors also suggests a role for MAIT cells in controlling progression of glioma. These findings are consistent with the reduction in tumor metastases after the antigen-dependent activation of MAIT cells in a mouse model of melanoma.^25,26^ However, the MR1 dependence of this effect is not yet clear.

Although GL261 cells could present antigen via MR1, as previously shown with astrocytes and microglia,^11^ the cells responsible for MR1 antigen presentation in the brain are unknown. Indeed, whether 5-OP-RU or other MAIT antigens are present in the brain TME is unknown, although it remains possible that MAIT cells are activated via cytokines during brain tumor growth, as they are during viral infections.^40^ Notably, a proposed MAIT cell role in maintaining meningeal barrier integrity is believed to be MR1-independent.^10^ Because *Mr1*^−/−^ mice are deficient in both MR1 and MAIT cells, the impact of MR1 could not be evaluated in our study. Notably, total MR1 expression could indicate immune cell infiltrate but may not correlate with MAIT cell activation in situ*,* which requires the expression of MR1 at the cell surface, whereas in the absence of antigen, MR1 is retained within the endoplasmic reticulum.^41^ Importantly, our findings demonstrate the importance of assessing MAIT cell tumor infiltration and activation alongside MR1 expression.

In our study, we analyzed MR1–5-OP-RU tetramer–reactive MAIT cells. It is possible that other MR1-reactive T cells (MR1Ts) are present and respond to unknown antigens. Although MR1T clones have been described in humans,^42^ they have not been characterized in mice, and their potential role in glioma will require the development of new detection tools.

Immune cells play a critical role in regulating brain homeostasis.^35^ γδ T cells and NKT cells have been implicated in murine models of ischemic stroke,^43^ and IL-17 production by γδ T cells is suggested to be important for brain homeostasis and functioning.^44^ A potential pathogenic role for IL-17–producing MAIT cells in patients with multiple sclerosis has been reported.^45^ In this study, we found that unconventional T cells, including MAIT cells, are highly tissue resident in the mouse brain, with MAIT17 and conventional Th17 cells displaying a higher degree of tissue residency than MAIT1/Th1 cells. Notably, both MAIT1 and MAIT17 cells remained predominantly tissue resident after systemic boosting. The local conversion of MAIT17 cells to a MAIT1 phenotype on infection or activation^46^ raises the intriguing possibility of MAIT cell modulation locally within the brain. Of interest, in both tumor-bearing brains and tumor tissues, a population of MAIT cells not expressing either Tbet or RORγt was detected. As MAIT cells have been reported to express GATA-3 in some contexts,^47^ it will be important in future to assess the presence of other MAIT subsets and their potential to drive anti-inflammatory or regulatory phenotypes during the immune response to tumors.

It is important to note that, despite being <1% of αβ T cells in the tumors, MAIT cells represented a much greater proportion of IL-17–producing αβ T cells. This increased after in vivo expansion; however, in mice with prior expansion of MAIT17 cells, MAIT cell cytokine production was reduced in tumors, perhaps reflecting functional exhaustion. Notably, the percentage of PD-1^+^ conventional T cells, but not MAIT cells, increased in tumor tissue. PD-1 may indicate either activation or exhaustion of tumor-infiltrating T cells.^48^ While T-cell exhaustion is not well characterized for MAIT cells, circulating MAIT cells from patients with cancers, including colon cancer, have been reported to be exhausted based on expression of PD-1, CTLA-4, Tim-3, and CD39.^49^ Notably, MAIT cell abundance has been linked to successful responses to anti–PD-1 therapies in patients with melanoma.^50^ Further analysis is needed to understand the activation state of MAIT cells in the brain. Notably, expression of CD69 by conventional T cells was significantly increased in tumors compared with tumor-bearing brains in WT but not *Mr1*^−/−^ mice, suggesting a minor role for MAIT cells in modulating other immune cell responses in GBM.

While the MR1-MAIT cell axis appears to affect survival after glioma induction in mice and humans, the mechanisms are not clear. There was no difference in tumor mass, lymphocyte infiltration, or cytokine production between WT and *Mr1*^−/−^ mice. Future studies should explore MAIT cell interactions within the TME, including their potential roles in stromal remodeling, angiogenesis, and modulation of microglial activity. Boosting MAIT cells alone before tumor induction did not improve survival in this rapidly progressing model, perhaps indicating that MAIT cells did not remain sufficiently activated after 28 days. Thus, the timing or maintenance of MAIT cell activation may be critical. Alternatively, GL261 tumor cells may exert suppressive effects on MAIT cells, as we observed a lower cytokine response of boosted MAIT cells within tumors and lack of PD-1 upregulation. Notably, our boosting protocols were not detrimental to survival and were well tolerated by the mice. This is consistent with intratumoral injection of CpG in patients with GBM, which was well tolerated and safe but showed limited clinical efficacy, and suggests that it is worthwhile to further explore MAIT cell modulation in glioma, for example, at different time points or in combination with checkpoint blockade.

Our results identified the activation state of MAIT cells as an important factor in their association with survival in glioma patient data, most notably in WHO grade III, and that MAIT cells provide a benefit in a mouse model of GBM. Our data reveal infiltration of GL261 tumors by MAIT cells, alterations to their phenotype within tumors, and a potential role in the conventional T-cell response. Furthermore, we demonstrate the effective modulation of MAIT cells in the brain using systemic protocols, providing platforms to explore their therapeutic potential. Moreover, these findings, together with Genome-Wide Association Study data linking MAIT cell–associated genes to susceptibility to cerebral tuberculosis,^16^ suggest that tissue-resident MAIT cells may play a broader role in immune surveillance of the brain, extending beyond cancer to include infectious diseases.

## Glossary

GBM: glioblastoma
MAIT: mucosal-associated invariant T
MR1: MHC class I–related molecule-1
MR1T: MR1-reactive T cell
NKT: natural killer T
PLZF: promyelocytic leukemia zinc finger
TCR: T-cell receptor
TME: tumor microenvironment
